# Optimization of Copper Electroforming Process Parameters Based on Double Hidden Layer BP Neural Network

**DOI:** 10.3390/mi12101157

**Published:** 2021-09-26

**Authors:** Feng Ji, Chao Chen, Yongfei Zhao, Byungwon Min

**Affiliations:** 1 College of Engineering, Mokwon University, Daejeon 35349, Korea; chenchao525@naver.com; 2 College of Xinglin, Nantong University, Nantong 226000, China; 3 College of Computer Science and Engineering, Sichuan University of Science and Engineering, Zigong 643033, China; 4 College of Mechanical Engineering, Nantong University, Nantong 226000, China; xiaoyamb@163.com

**Keywords:** double hidden layer, BP neural network, electroforming, optimization

## Abstract

In order to optimize the pulse electroforming copper process, a double hidden layer BP (back propagation) neural network was constructed. Through sample training, the mapping relationship between electroforming copper process conditions and target properties was accurately established, and the prediction of microhardness and tensile strength of the electroforming layer in the pulse electroforming copper process was realized. The predicted results were verified by electrodeposition copper test in copper pyrophosphate solution system with pulse power supply. The results show that the microhardness and tensile strength of copper layer predicted by “3-4-3-2” structure double hidden layer neural network are very close to the experimental values, and the relative error is less than 2.82%. In the parameter range, the microhardness of copper layer is between 100.3~205.6 MPa and the tensile strength is between 165~485 MPa. When the microhardness and tensile strength are optimal, the corresponding range of optimal parameters are as follows: current density is 2–3 A·dm^−2^, pulse frequency is 1.5–2 kHz and pulse duty cycle is 10–20%.

## 1. Introduction

In the electroforming process, the method of deposited layer formation is important as it is used to prepare metal parts with special shapes or molds with high-precision requirements [1,2,3]. The electroforming process relies on the electrons from metal ions stacked on the cathode surface one by one, and its theoretical accuracy can reach the ion level. It is widely used in the preparation of microstructural parts with small and complex structures on the surface. Traditional electroforming technology has problems such as uneven distribution of electric field intensity, hydrogen evolution and difficult mass transfer at the microstructure of parts. The electroforming layer is prone to quality defects such as pinholes, pits and cavities, and the deposition thickness is uneven, which means that it cannot completely copy the whole microstructure, affecting the forming quality of microstructure parts [4,5]. When the complex structure is copied by DC power electroforming, the nodulation occurs on the surface and edge of the electroforming layer. At the same time, the grain size and surface roughness of the deposited layer appear, and the performance index of the electroforming layer cannot meet the actual demand. Through a large number of experimental studies, many researchers have found that electroforming with pulse power supply is an important means to improve the quality of the deposited layer [6,7,8]. 

In pulse electroforming, the electrode process changes from a DC process to a periodic pulse process. The pulse power supply affects the electrode process, thus affecting the physical properties and quality of the deposited layer. Pulse electroforming can supplement the concentration of metal ions in the diffusion layer, significantly reduce the concentration polarization and produce higher electrochemical polarization, so as to refine the grain and improve the density of the deposited layer. The thickness of the diffusion layer is reduced due to the intermittent formation around the diffusion layer, increasing the cathode limiting current density, the metal crystal morphology and the growth mode during electroforming, which are closely related to the cathodic polarization overpotential. Increasing the overpotential can make the grain finer and the deposited layer compact. In pulse electroforming, the uniformity of the deposited layer is affected by the distribution of current density and the transmission of electrolyte components. Intermittent energization can buffer the ion concentration on the cathode surface, greatly reduce the difference in the thickness of the deposited layer caused by the uneven transmission of electrolyte components and improve the uniformity of the deposited layer.

Yuan Xuetao ascertained that the grain size of deposits with fine appearance can only be refined down to about 100 nm by changing the pulse parameters [9]. Fafeng Xia used the pulse electrodeposition (PED) technique, which showed that the contents of AlN nanoparticles increased with increasing density of pulse current and on-duty ratio of pulse current [10]. S.A.Lajevardi investigated the effects of pulse electrodeposition parameters on the properties of nickel–titania composite coatings electrodeposited from a nickel Watts-type bath [11]. M.E. Bahrololoom found that the nickel–alumina coatings deposited by pulse current were harder than the same coatings produced from the same bath with DC current. Similarly to their hardness, utilization of pulse currents was found to increase the wear resistance of these coatings [12]. Cui, W. et al generated Ni-doped TiN coatings on 45 steel surfaces by ultrasound assisted pulse electrodeposition from reformative Watt nickel-based electrolytes [13].

In the current electroforming research process, a large number of experiments need to be carried out to obtain the relationship between electrodeposition process parameters and coating properties, which greatly affects the efficiency of product development in experimental and industrial production, and therefore consumes a lot of manpower and time costs. The artificial neural network has a good self-learning function and can efficiently find the optimal parameters of the electroforming process. It is widely used in the fields of intelligent machining, signal processing and optimal combination.

Gao Xiu-Hua et al. used an artificial neural network to predict the target performance of the casting layer, obtain the best parameters and solve a series of problems in the actual production of the electroforming industry [14]. Subramanian, K. studied electrodeposit plating parameters of copper–tin over a mild steel substrate and developed a model using artificial neural networks (ANN). The ANN model is compared with the conventional mathematical regression model for analysis [15]. Minzhen Jiang obtained the optimum conditions for fabricating Ni-TiN nanocoatings based on the greatest hardness of Ni-TiN deposits via a three-layer backward propagation model [16]. Hui Wang used a back-propagation neural network model with 3-8-1 structure to predict the wear resistance of an ultrasonically electrodeposited Ni-SiC nanocoating [17]. Youjun Xu used a feed-forward, multilayer perceptron artificial neural network (ANN) model with eight hidden layers and 12 neurons to predict the corrosion behavior of Ni–SiC composite coatings deposited by ultrasonic electrodeposition [18]. Sun WC prepared Ni-TiC composite coatings on a substrate of aluminum alloy by pulse electrodeposition and established a three-layer back propagation artificial neural network with Lavenberg-Marquardt algorithm by MATLAB, which was used to train the network and predict orthogonal experimental data [19].

In this paper, the optimal parameters of the pulse electroforming copper process were studied. The mapping relationship between the input parameters and the output parameters of microhardness and tensile strength was established by using a double hidden layer back-propagation neural network. Through model training, the linear inseparable problem under the electroforming copper processing model was solved by using the multi-dimensional functional mapping ability and self-learning ability of multi-layer perceptron. Finally, the process parameters of the optimal performance of electroformed copper were predicted. 

The remainder of the paper is structured as follows. Section 2 provides details about electroforming copper and the double hidden layer BP neural network method. The network prediction results and test results are given in Section 3 and the paper ends with a conclusion in Section 4.

## 2. Methods

### 2.1. Copper Electroforming Method 

**Figure 1:**
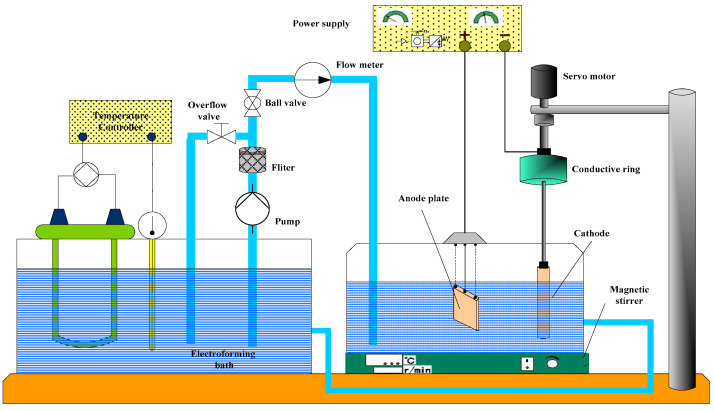
Schematic diagram of electroforming system.

The self-developed electroforming copper system is shown in the Figure 1, including electroforming liquid circulation system, electroforming liquid temperature control system, fixture system and power supply system. The anode plate is made of phosphor copper. The cathode shape is a metal tube with a diameter of 6 mm and a length of 50 mm, a section of which is as shown in Figure 1. The rest cathode immersed in the solution is insulated. The distance between anode and cathode was fixed at 40 mm.

Pyrophosphate electroforming solution has good dispersion and coverage ability, and it is not easy to produce hydrogen in the electroforming process. In this paper, pyrophosphoric acid solution was used for copper electroforming. During the experiment, we adjusted the pH value of the solution with ammonia and ammonium citrate. The specific formula of the solution is shown in the Table 1. The specific experimental parameters involved in this paper are shown in the Table 1. The microhardness of the electroformed copper layer was measured by the TMVS-1 digital microhardness tester from Beijing Time High Technology Co., Ltd. (Beijing, China). The tensile strength was tested by CMT5105 universal testing machine from METS industrial systems Co., Ltd. (Wuhan, China).

According to Faraday’s first law and second law, we deduced that the thickness of metal layer deposited on the cathode surface $\delta_{t}$ is:

(1) $$\delta_{t}=\frac{M}{\rho s}=\frac{100KIt\eta_{1}}{\rho s}=\frac{100Kdt\eta_{1}}{\rho }$$

In Formula (1), ρ is the metal density (g/cm^3^), *I* is the circulating current (A), *s* is the cathode deposition area (dm^2^), η_1_ is the current efficiency and *d* is the current density. The thickness of the metal deposition layer $\delta_{t}$ is directly proportional to the current density *d*. Frequency *f* and duty cycle of pulse power supply *γ* directly affect the quality of the electroforming layer.

The pulse frequency affects the pulse period directly. A reasonable pulse frequency is conducive to reducing the concentration polarization, promoting the grain refinement of the copper layer and improving the compactness of the copper layer. An appropriate pulse duty cycle is beneficial to interrupt the continuous growth of grains, reduce the critical nucleation radius of grains and make the microstructure of copper layer compact. Therefore, the aim of this paper was to build a nonlinear mapping model between the input parameters of current density, pulse frequency and pulse duty cycle and output parameters of microhardness and tensile strength, which affect the electroforming results.

**Figure 2:**
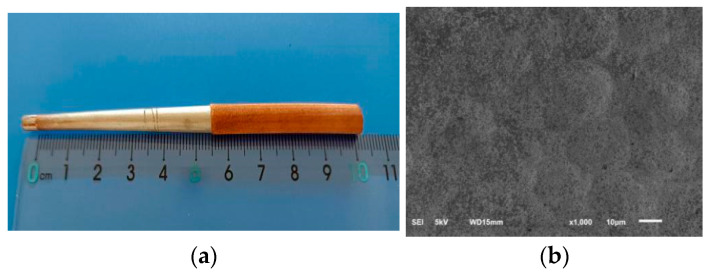
Experimental results of the sample with pyrophosphoric acid solution. (**a**) morphology of the sample, (**b**) SEM image of copper layer on sample surface.

Figure 2 shows the pictures of test results of electroforming copper with pyrophosphoric acid solution. Figure 2a is the morphology of the sample, and Figure 2b is the SEM image of copper layer on sample surface.

### 2.2. Double Hidden Layer BP Network Model

In this paper, the microhardness and tensile strength in the copper electroforming process were trained and predicted based on a BP neural network. Increasing the number of nodes of the single hidden layer alone cannot improve the prediction ability of the neural network for copper electroforming process parameters. In this paper, a method of adding a second hidden layer is proposed, which uses the multi-dimensional function mapping ability and self-learning ability of multi-layer perceptrons. When dealing with the linear inseparable problem under the electroforming copper processing model, the double hidden layer BP neural network is superior. 

#### 2.2.1. Model Design

The BP neural network has the characteristics of signal forward calculation and error back propagation. The multilayer feedforward network, composed of an input layer, hidden layer and output layer, is gradually optimized by a back-propagation algorithm, and the weight of each perceptron is continuously adjusted according to the minimum loss function until the training goal is reached. 

**Figure 3:**
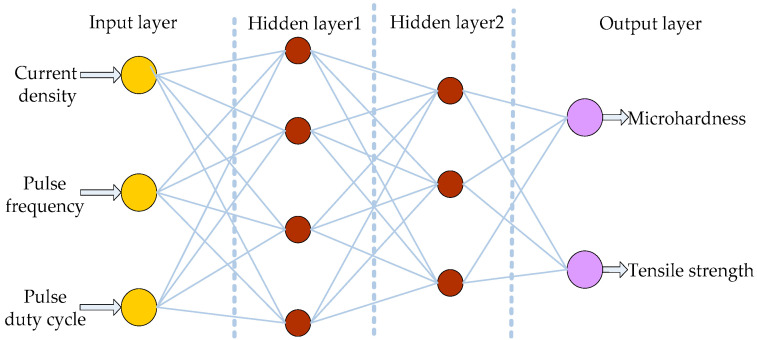
Double hidden layer network structure.

In the network model of copper electroforming process parameter optimization, three parameters that most affect the results of copper electroforming were selected as input—*d*, *f* and *γ* correspond to the nodes of the input layer. respectively. The three components have different physical meanings with small correlations, which can be detected and extracted. Microhardness *Hv* and tensile strength *Rm* were selected as the output items of BP network. The double hidden layer network constructed in this paper is shown in Figure 3. In the BP network, the first hidden layer has 4 nodes and the second hidden layer has 3 nodes.

#### 2.2.2. Model Training Description 

In this paragraph, the variables and parameters of double hidden layer BP neural network are defined, as shown in Table 2. Before model training, the samples need to be preprocessed to remove the extremely similar parameters in the sample input items, so that the double hidden layer BP network has better approximation and generalization ability. The transfer function in network model training is a unipolar sigmoid function, which has a good effect. The sigmoid function is: f(x) = (1 + e^−x^)^−1^.

The training steps of network model are as follows: 

Step 1: Initialization sample counter *p*, training times counter *q*, weight matrix W·V, learning rate *η*_2_, error accuracy ε = 10^−6^, threshold *b*.

Step 2: Input samples and calculate the output of each layer, $y_{j}^{1}$, $y_{j}^{2}$, $o_{k}$ are calculated by Equations (2)–(4):

(2) $$y_{j}^{1}=f\left(\overset{n}{\underset{i=1}{\sum }}v_{ij}x_{i}+b\right)$$

(3) $$y_{j}^{2}=f\left(\overset{m}{\underset{j=1}{\sum }}w_{ij}y_{j}^{1}+b\right)$$

(4) $$o_{k}=f\left(\overset{m}{\underset{k=1}{\sum }}w_{jk}y_{j}^{2}+b\right)$$

Step 3: Calculate the network output error. Assuming that there are *p* pairs of training samples, the root mean square error is used as the total error of the network by Equation (5):

(5) $$E=\sqrt{\frac{1}{2}\overset{P}{\underset{p=1}{\sum }}\overset{n}{\underset{k=1}{\sum }}{\left(d_{k}^{p}-o_{k}^{p}\right)}^{2}}$$

Step 4: Calculate the error signal of each layer.$\;\delta_{k}^{o}$ $\delta_{j}^{h_{1}}\delta_{j}^{h_{2}}\;$are calculated by Equations (6)–(8):

(6) $$\delta_{k}^{o}=\left(d_{k}-o_{k}\right)o_{k}\left(1-o_{k}\right)$$

(7) $$\delta_{j}^{h_{2}}=\overset{n}{\underset{k=1}{\sum }}\delta_{k}^{o}w_{jk}y_{j}^{2}\left(1-y_{j}^{2}\right)$$

(8) $$\delta_{j}^{h_{1}}=\overset{n}{\underset{k=1}{\sum }}\delta_{j}^{h_{2}}w_{ij}y_{j}^{1}\left(1-y_{j}^{1}\right)$$

Step 5: Adjust the weight of each layer. After the error signal is obtained, the weight of each layer is inversely adjusted by the error until the network performance reaches the preset accuracy. $\Delta w_{jk}$ $\Delta w_{ij}$ $\Delta v_{ij}$ are calculated by Equations (9)–(11):

(9) $$\Delta w_{jk}=\eta_{2}\delta_{k}^{o}y_{j}^{2}=\eta_{2}\left(d_{k}-o_{k}\right)o_{k}\left(1-o_{k}\right)y_{j}^{2}$$

(10) $$\Delta w_{ij}=\eta_{2}\delta_{j}^{h_{2}}y_{j}^{1}=\eta_{2}\left(\overset{m}{\underset{k=1}{\sum }}\delta_{k}^{o}w_{jk}\right)y_{j}^{2}\left(1-y_{j}^{2}\right)y_{j}^{1}$$

(11) $$\Delta v_{ij}=\eta_{2}\delta_{j}^{h_{1}}x_{i}=\eta_{2}\left(\overset{m}{\underset{j=1}{\sum }}\delta_{j}^{h_{2}}w_{ij}\right)y_{j}^{1}\left(1-y_{j}^{1}\right)x_{i}$$

Step 6: Check whether all samples are trained once. If *p* < *P*, the counters *p* and *q* increase by 1 and return to step 2. Otherwise, enter step 7. 

Step 7: Check whether the total network error reaches the accuracy. When *E* < ε, the training ends. Otherwise, *p* is set to 1 and enter step 2 to start a new round of training for the sample. 

## 3. Results and Analysis

### 3.1. Sample Pretreatment

Firstly, the samples were reduced. At the beginning of the processing of electroformed copper processing test samples, 137 samples similar to the expected experiment were selected, normalized with sigmoid function and the data of different dimensions were changed to [0, 1] or [−1, 1] for the input samples. Table 3 shows some samples. Normalization can avoid the saturation of neuron output caused by a continuous value that is too large and slow down the convergence speed. In this paper, it was transformed by Equation (12).

(12) $$x^{*}=\frac{x-x_{min}}{x_{max}-x_{min}}$$

x* is the normalized sample, *x* is the original value, *x_min_* and *x_max_* are the minimum and maximum values, respectively. In the forward propagation learning of the signal in the network, the difference between the output signal and the teacher signal was compared, the error signals of the output layer and the double hidden layer were inversely calculated according to the error value, and the corresponding weights were adjusted layer by layer.

### 3.2. Analysis of Network Model Experiment Results

Due to the lack of nonlinear mapping ability of a single hidden layer network in electroforming copper process optimization, increasing the number of nodes in a single hidden layer cannot improve the performance of the network. In this paper, it was determined that adding a hidden layer can improve the mapping ability of the artificial network in terms of parameters such as current density *d*, pulse frequency *f*, pulse duty cycle *γ*, microhardness *Hv* and tensile strength *Rm*. The node design in the double hidden layer network needs to avoid overfitting or increasing the training time of samples.

In this paper, the trial-and-error method was used to determine the number of neurons in the double hidden layer. By detecting the network performance of different numbers of nodes in the double hidden layer for the same sample, the optimal number of nodes was obtained. Firstly, the trial-and-error experiment was carried out on the single hidden layer, for which the optimal number of nodes in the network was seven and the relative error of the prediction result was 0.112. Then, eight groups of experiments were designed based on the characteristics of the double hidden layer network.

Based on the number of nodes in a single hidden layer, two principles should be followed in the construction of a double hidden layer: 1. The total number of nodes in a double hidden layer should not be greater than the number of nodes in a single hidden layer; 2. The number of nodes in the second hidden layer should be less than that in the first layer. As shown in Table 4, the number of double hidden layer nodes in the six groups of experiments was not greater than 7, and two groups of experiments with more than seven nodes were set for comparison. According to the relative error of the combination of multiple nodes in the hidden layer in the prediction results, the optimal structure of the double hidden layer network was determined as: 3-4-3-2. The first layer of the double hidden layer has four nodes and the second layer has three nodes. This structure had a good performance in the prediction of the network. The experimental results are shown in Table 4.

The running time in Table 4 is the time when the network tends to be stable. Generally, when designing a more complex network, it is necessary to take the cross-value of the network error change curve of the model in the training sample and the test sample. At this time, the error in the training sample tends to be smaller, while the error in the test set tends to be larger. If only the error value is considered, there may be overfitting, which leads to strong performances in the training samples and insufficient results in the test samples, affecting the generalization ability of the model. There was no fitting phenomenon in this experiment and the network performance was good.

### 3.3. Model Prediction Results

Using the trained copper electroforming process neural network prediction model, 12 groups of process parameters were used as the input of neural network to predict the electroforming results. The output values of neural network and the actual electroforming results of experiments are shown in Table 5. The values of current density were 1 A, 2 A, 3 A and 4 A, the values of pulse frequency were 0.5 kHz, 1 kHz, 1.5 kHz, 2 kHz, 2.5 kHz and 3 kHz, the values of pulse duty cycle were 10%, 20%, 40%, 60% and 80%, the distribution of output parameters was reasonable, the microhardness of copper layer was between 100.3~205.6 MPa and the tensile strength was between 165~485 MPa. The predicted values of the electroforming copper process neural network in Table 5 were basically consistent with the actual values, the neural network had good generalization ability and the relative error was less than 2.32%. The predicted results of neural network and the actual measurement results showed that the corresponding process conditions for the best microhardness and tensile strength are as follows: the current density *d* is 2 A·dm^−2^, the pulse frequency *f* is 2 kHz and the pulse duty cycle *γ* is 10%.

### 3.4. Experimental Verification

**Figure 4:**
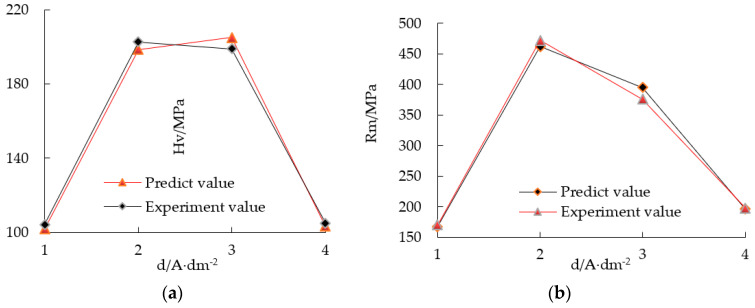
Comparison between the experimental and predicted values with different current density. (**a**) Microhardness and (**b**) tensile strength.

**Figure 5:**
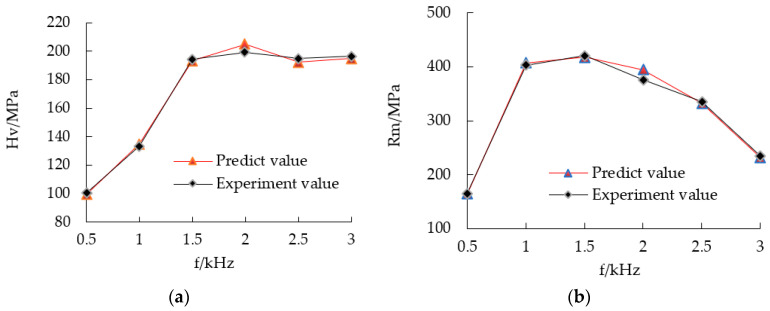
Comparison between the experimental and predicted values with different frequencies. (**a**) Microhardness and (**b**) tensile strength.

In the experiment of electroformed copper, the experiment was carried out according to the input parameters in Table 5. Neural network prediction and physical experiments showed that with the change of pulse current density, frequency and duty cycle, the microhardness and tensile strength of the electroforming layer changed significantly, mainly in the following aspects:

(1) Neural network prediction and experiments showed the effect of current density on microhardness and tensile strength. The microhardness of the electroformed layer decreased with the increase of current density. As shown in Figure 4a, when the average pulse current density was 2 A·dm−2, the microhardness of copper layer was the highest, reaching 202.7 MPa, which was close to the value when the current density was 3 A·dm−2. When the average pulse current density increased to 4 A·dm−2, the microhardness of the copper layer decreased to 105 MPa. The tensile strength of the electroformed copper layer reached 472 MPa at 2 A·dm−2, and it decreased with the increase of current density, as shown in Figure 4b. When the average current density increased from 1 A·dm−2 to 2 A·dm−2, the grain size became larger. According to the fine grain strengthening theory, the hardness and tensile strength of the material are inversely proportional to the grain size. Therefore, the microhardness and tensile strength of the copper layer will continue to decline.

(2) Neural network prediction and experiments showed the effect of pulse frequency on microhardness and tensile strength. With the increase of pulse frequency, the microhardness of electroformed copper layer increased first and then stabilized, as shown in Figure 5a. When the pulse frequency was 0.5 kHz, the minimum microhardness of electroformed copper layer was 100.3 MPa. As the pulse frequency continued to increase, the microhardness of the electroforming layer also increased, and reached the peak at 2 kHz. 

The tensile strength of copper layer first increased and then decreased with the increase of frequency. In the frequency range of 0.5–2 kHz, the tensile strength of the copper layer increases from 165 MPa to 421 MPa, as shown in Figure 5b. The tensile strength of the electroforming layer decreases greatly with the increase of pulse frequency. Therefore, the optimal selection range of frequency is 1.5–2 kHz.

**Figure 6:**
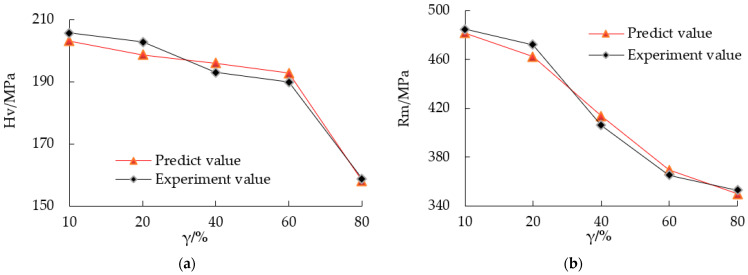
Comparison between the experimental and predicted values with different duty cycles. (**a**) Microhardness and (**b**) tensile strength.

Neural network prediction and experiments showed the effect of pulse duty cycle on microhardness and tensile strength. The microhardness of electroformed copper layer gradually increased with the decrease of duty cycle, and reached 205.6 MPa when the duty cycle was 10%, as shown in Figure 6a. When the duty cycle was 10%, the surface morphology of the copper layer was the most smooth and flat, and the grain size was the smallest and most uniform. According to the fine grain strengthening theory, the microhardness of the electroforming layer is the largest, and the results are consistent.

The tensile strength of the electroforming layer decreased with the increase of the duty cycle. When other conditions were constant and the duty cycle increased from 10% to 80%, the tensile strength of the electroforming layer decreased from 485 MPa to 353 MPa, as shown in Figure 6b. The analysis showed that the change of duty cycle changed the grain size and microstructure of electroformed copper layer, resulting in the change of strength of the electroformed copper layer, and its change trend was consistent with the change trend of grain size. When the duty cycle exceeded 20, the hardness decreased and tensile strength sharply decreased. Therefore, the optimal selection range of the duty cycle is 10–20%.

**Figure 7:**
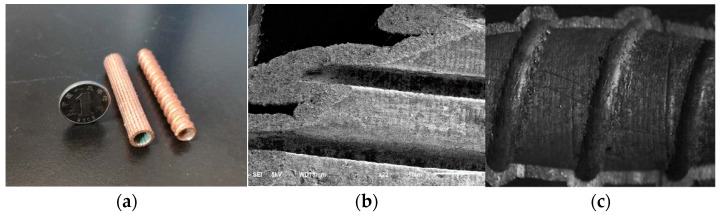
Microstructure of rotating inner wall prepared by electroforming. (**a**) The samples of the cathode, (**b**) The intenal morphology of one sample, (**c**) The internal morphology of the other sample.

In this paper, the pulse average current density of 2–3 A·dm−2, duty cycle of 10–20% and frequency of 1.5–2 kHz were selected as the range of optimal electroforming process parameters to ensure better surface quality and mechanical properties. The sample generated by electroforming with the parameters in the optimal range is shown in Figure 7a. Figure 7b,c shows the internal morphology of the two samples. The rotary inner wall micro groove structure was prepared by electroforming, as shown in Figure 7b, and the micro groove depth is about 955 μm. The width is about 455 μm. Figure 7c shows the flow line structure of the rotating inner wall prepared by electroforming, with a depth of about 500 μm.

## 4. Conclusions

In this paper, the predicted results and experimental results of a double hidden layer BP neural network were used to obtain the optimum tensile strength and microhardness of pulse cast copper. The range of optimal electroforming process parameters are as follows: current density is 2–3 A·dm^−2^, pulse frequency is 1.5–2 kHz and pulse duty cycle is 10–20%. Under these conditions, a uniform and dense copper layer can be obtained.

The relationship between electroforming copper process parameters and tensile strength and microhardness is nonlinear and complex, and there is no specific correlation expression. Double hidden layer BP neural network has good nonlinear mapping ability and generalization ability, and has great advantages over other empirical formula methods. It carries out training and learning by optimizing the number of nodes in the double hidden layer of the network and identifies the complex relationship between the input and output of electroforming copper process, which provides an innovative way to solve the optimization problem of electroforming copper process parameters.

Double hidden layer BP neural network can comprehensively consider the influence of electroforming copper process parameters on deposition results. The predicted tensile strength and microhardness were close to the actual experimental results, and the training accuracy was high. It has a good popularization ability in the field of intelligent electroforming.

## Figures and Tables

**Table 1:** Chemical composition of the electroforming bath.

Solution Composition or Process Conditions	Content or Parameter
Cu_2_P_2_O_7_ (g/L)	65
K_4_P_2_O_7_·3H_2_O (g/L)	300
NH_3_·H_2_O (ml/L)	2.5
((NH_4_)_2_HC_6_H_5_O_7_) (g/L)	22
pH	8.0–8.8
Temperature (°C)	50–55

**Table 2:** Meaning of symbols.

Symbol	Meaning	Symbol	Meaning
*X*	Input layer vector	*h*_2_	Second layer of hidden layer
*O*	Output layer vector	$y_{j}^{1}$	Output of *h*_1_
*d*	Output layer expectation vector	$y_{j}^{2}$	Output of *h*_2_
*V_ij_*	Weight between input layer and *h_1_*	$\delta_{k}^{o}$	Error singal of output layer
*W_ij_*	Weight between *h*_1_ and *h*_2_	$\delta_{j}^{h1}$	Error singal of *h*_1_
*W_jk_*	Weight between *h*_2_ and output layer	$\delta_{j}^{h_{2}}$	Error singal of *h*_2_
*h_1_*	First layer of hidden layer	ε	Accuracy of network
*p*	Sample counter	*η_2_*	Learing rate
*q*	Training times counter	*b*	Threshold

**Table 3:** Partial sample.

Name	Import			Teacher	
	*d*/A·dm^−2^	*f*/kHz	*γ*/%	*Hv*/MPa	*Hm*/MPa
1	1	2	60	156.3	190
2	2	1.5	20	193.5	418
3	2	3	40	186.1	381
4	3	2	40	190.2	356
5	3	3	60	178.2	323

**Table 4:** Network performance with different number of neurons.

Number of Nodes in *h*_1_	Number of Nodes in *h*_2_	Network Relative Error	Running Times/s
6	1	0.102	4.3
5	2	0.095	4.1
4	3	0.022	2.9
4	2	0.049	3.3
3	4	0.077	4.2
7	1	0.109	3.4
5	3	0.105	3.7
3	3	0.085	3.1

**Table 5:** The comparison of BP NN prediction with experimental results.

Name	Network Input			Network Output		Experiment Results	
	*d*/A·dm^−2^	*f*/kHz	*γ*/%	*Hv*/MPa	*Rm*/MPa	*Hv*/MPa	*Rm*/MPa
1	1	2	20	101.90	167.62	104.3	170
2	2	2	20	198.65	462.09	202.7	472
3	3	2	20	204.97	394.80	199	376
4	4	2	20	103.43	195.82	105	198
5	2	2	10	203.13	481.61	205.6	485
6	2	2	40	195.90	413.72	193	406
7	2	2	60	192.75	369.38	189.9	365
8	2	2	80	158.11	349.82	158.9	353
9	3	0.5	20	99.20	163.85	100.3	165
10	3	1	20	135.00	407.03	133.4	403
11	3	1.5	20	193.33	418.47	194.3	421
12	3	2.5	20	192.27	333.31	195	336
13	3	3	20	195.03	232.42	196.4	235
